# Drought Resilience in Oil Palm Cultivars: A Multidimensional Analysis of Diagnostic Variables

**DOI:** 10.3390/plants13121598

**Published:** 2024-06-08

**Authors:** Cristihian Bayona-Rodríguez, Hernán Mauricio Romero

**Affiliations:** 1 Colombian Oil Palm Research Center—Cenipalma, Oil Palm Biology and Breeding Research Program, Bogotá 11121, Colombia; cbayona@cenipalma.org; 2 Department of Biology, Universidad Nacional de Colombia, Bogotá 11132, Colombia

**Keywords:** ecophysiology, reactive oxygen species, antioxidant response, breeding, water deficit, *Elaeis guineensis*

## Abstract

Water scarcity is a significant constraint on agricultural practices, particularly in Colombia, where numerous palm cultivators rely on rainfed systems for their plantations. Identifying drought-tolerant cultivars becomes pivotal to mitigating the detrimental impacts of water stress on growth and productivity. This study scrutinizes the variability in drought responses of growth, physiological, and biochemical variables integral to selecting drought-tolerant oil palm cultivars in the nursery. A comprehensive dataset was compiled by subjecting seedlings of eleven cultivars to four soil water potentials (−0.05 MPa, −0.5 MPa, −1 MPa, and −2 MPa) over 60 days. This dataset encompasses growth attributes, photosynthetic parameters like maximum quantum yield and electron transfer rate, gas exchange (photosynthesis, transpiration, and water use efficiency), levels of osmolytes (proline and sugars), abscisic acid (ABA) content, as well as antioxidant-related enzymes, including peroxidase, catalase, ascorbate peroxidase, glutathione reductase, and superoxide dismutase. Principal Component Analysis (PCA) elucidated two principal components that account for approximately 65% of the cumulative variance. Noteworthy enzyme activity was detected for glutathione reductase and ascorbate peroxidase. When juxtaposed with the other evaluated cultivars, one of the cultivars (IRHO 7001) exhibited the most robust response to water deficit. The six characteristics evaluated (photosynthesis, predawn water potential, proline, transpiration, catalase activity, sugars) were determined to be the most discriminant when selecting palm oil cultivars with tolerance to water deficit.

## 1. Introduction

Oil Palm (*Elaeis guineensis*) is the most productive oil crop in the world, with the highest human consumption [1]. The use of palm oil derivatives is constantly increasing, and today, half of the products in supermarkets are palm oil-based [2]. This scenario has stimulated producers’ interest in improving the oil’s yield and quality, closing production gaps through agronomic practices, the modernization of agricultural processes, and the genetic improvement of cultivars [3]. The classical plant breeding process in oil palm requires many years of monitoring and evaluating oil palm with a multidisciplinary and collaborative research approach [4]. However, with the prediction of more frequent and severe extreme events, such as drought phases or heat waves driven by climate change [5], new approaches are required for improving oil palm to accelerate the development of cultivars with desirable characteristics [6,7]. These improvements will lead to increased yields in existing plantations and will pave the way to minimizing the undesired ecological impacts of oil palm agriculture while improving its social benefits [8].

Colombia ranks fourth among the producing countries, with approximately 600,000 ha planted in four oil palm-growing zones [9]. In these zones, dry seasons occur at certain times of the year, lasting from 3 to 8 months, depending on weather conditions [10]. Except for the western zone, these dry periods can severely impact oil palm production, generally because of the variation in the proportion of male and female inflorescences in periods of water deficit [11]. Different studies have shown a marked sensitivity of oil palm genotypes to drought [12], indicating that water stress causes a significant decrease in photosynthesis and transpiration rates and delayed growth [13,14,15], fluctuations in biochemical and molecular responses [16], and, consequently, a reduction of more than 30% in production [17]. These negative implications motivate breeding programs to develop new cultivars with drought tolerance and increased production and disease tolerance.

The results of oil palm studies focusing on the behavior of the plants in response to water deficit have covered different perspectives, showing a differential response with morphological, physiological, and biochemical strategies that give some cultivars characteristics that allow them to obtain different degrees of drought tolerance. Growth parameters [18], gas exchange [19,20], and biochemical responses regarding the osmolytes and antioxidant enzymes [16] are among the variables most related to drought tolerance in oil palm. Although each group of researchers in the studies above has addressed one or two groups of response variables, the final objective is to generate information that helps to identify tolerant genotypes, understand the tolerance mechanisms, and use them to breed new water deficit-tolerant cultivars.

This work aimed to use growth parameters, gas exchange, chlorophyll fluorescence, protective osmolytes, and antioxidant enzyme response to understand their contribution to drought tolerance in oil palm seedlings. The idea would be to generate an early selection tool for the breeding program. Additionally, the results will allow for discriminating cultivars planted in water-limiting areas, substantially improving the genotype × environmental response, translating into productivity and social benefits.

## 2. Results

### 2.1. Growth Parameters

**Figure 1:**
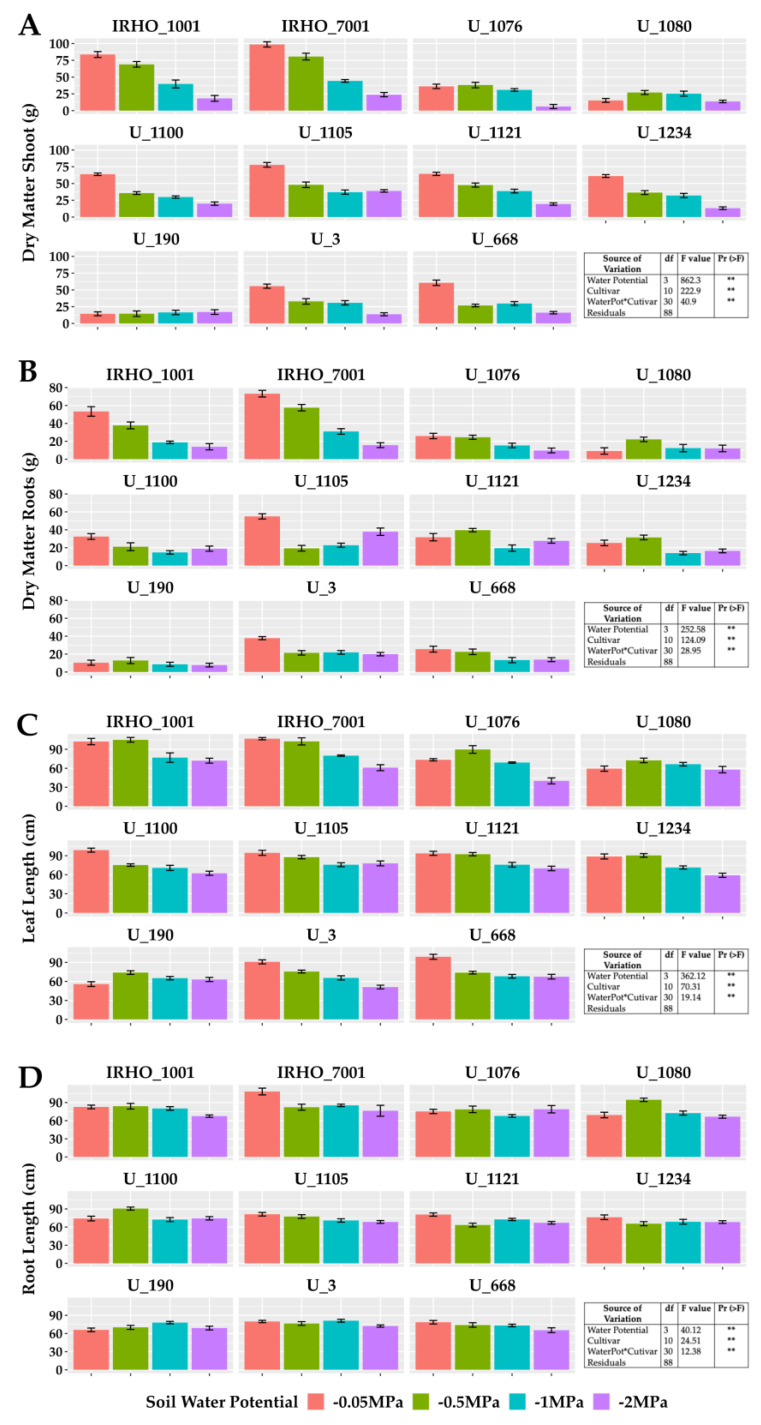
Growth variables in seedlings of 11 oil palm (*Elaeis guineensis*) cultivars subjected to four soil water potentials (−0.05 MPa, −0.5 MPa, −1 MPa, and −2 MPa) during 60 days of treatment. (**A**) Shoot dry matter. (**B**) Root dry matter. (**C**) Leaf length. (**D**) Root length. Each box corresponds to the mean ± SD. (n = 15). The tables in each panel correspond to the two-way ANOVA with significance levels. ** highly significantly different *p* ≤ 0.01.

Figure 1 shows the results obtained for biomass variables and elongation of the aerial part (bulb plus leaves) and roots. A significant difference was found between treatments for the two variables, showing a negative effect of water deficit on these organs in the 11 cultivars; as water stress became stronger, plants generally showed slower growth. However, depending on the response variable, such as root biomass (Figure 1B), cultivars responded heterogeneously to moisture levels; that is, plant growth in some cultivars was directly affected by the four soil water potentials (IRHO 1001, IRHO 7001, U 1076), others showing the same degree of water deficit (U 3, U 668) and other cultivars without a clear pattern. The cultivar IRHO 1001 had the most significant decrease (80%) in root biomass among plants grown under field capacity conditions (−0.05 MPa) and severe water deficit (−2 MPa). The least affected was U 1121, with 13%.

**Figure 2:**
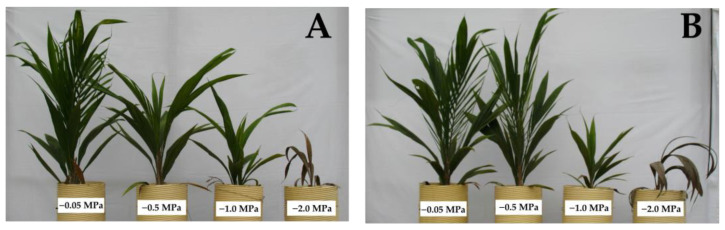
Physical appearance of two oil palm (*Elaeis guineensis*) cultivars subjected to four soil water potentials (−0.05 MPa, −0.5 MPa, −1 MPa, and −2 MPa) during 60 days of treatment. (**A**) IRHO 7001 cultivar. (**B**) U-1221 cultivar.

Regarding the shoot biomass (Figure 1A), despite presenting similar behavior as the root biomass, there was a higher proportion of cultivars that responded directly to the water stress levels (IRHO 7001, U 1100, U 3, U 668), with U 1076 being the most affected (83%) and U 1080 the lowest (10%). Regarding the length variables, the aerial portion (Figure 1C) was most affected, with reductions of 50% in size, making the water deficit visible, and with short palms and, in the case of more severe stress, a tendency for the leaves to wilt (Figure 2). Regarding root length (Figure 1D), smaller reductions of close to 30% were seen in the most affected cultivars.

### 2.2. Physiological Parameters

**Figure 3:**
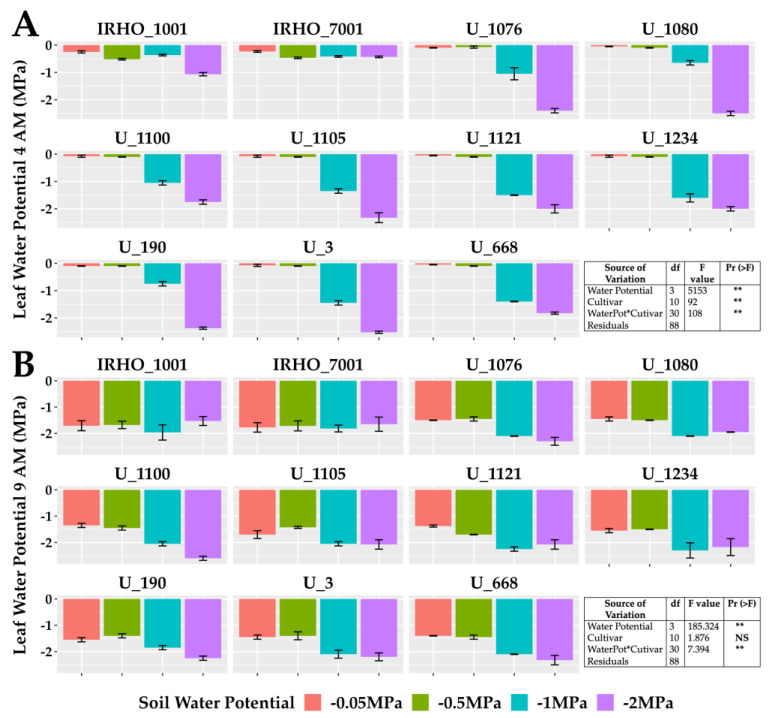
Leaf water potential of seedlings of 11 oil palm (*Elaeis guineensis*) cultivars subjected to four soil water potentials (−0.05 MPa, −0.5 MPa, −1 MPa, and −2 MPa) during 60 days of treatment. (**A**) Predawn (4:00 AM) leaf water potential. (**B**) Actual (9: 00AM) leaf water potential. Each box corresponds to the mean ± SD. (n = 15). The tables in each panel correspond to the two-way ANOVA with significance levels. NS: not significant; ** highly significantly different *p* ≤ 0.01.

The predawn leaf water potential (Ψleaf) (Figure 3A) is one of the best indicators of the plant’s water status and reflects the magnitude of the stress in the plants. All cultivars showed more negative Ψleaf under the most severe stress (−2 MPa), with values as low as −2.5 MPa, and the least affected cultivar was IRHO 7001. The actual Ψleaf gap (Figure 3B) between treatments was smaller in all cultivars.

**Figure 4:**
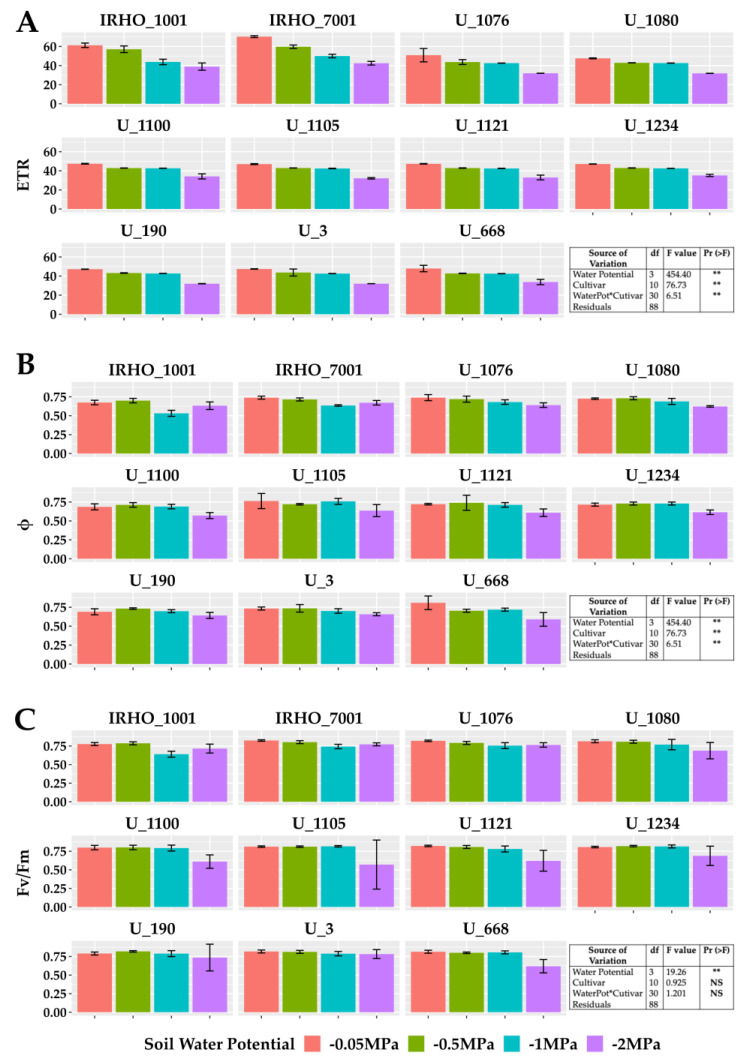
Chlorophyll A fluorescence parameters of seedlings of 11 oil palm (*Elaeis guineensis*) cultivars subjected to four soil water potentials (−0.05 MPa, −0.5 MPa, −1 MPa, and −2 MPa) during 60 days of treatment. (**A**) ETR, electron transfer rate. (**B**) Φ, quantum yield of PSII. (**C**) Fv/Fm, maximum quantum yield of PSII. Each box corresponds to the mean ± SD. (n = 15). The tables in each panel correspond to the two-way ANOVA with significance levels. NS: not significant; ** highly significantly different *p* ≤ 0.01.

Among the variables related to chlorophyll fluorescence (Figure 4), the PSII (photosystem II) maximum quantum yield of photochemical efficiency (Fv/Fm), associated with dark-adapted measurements (Figure 4C), showed a stable behavior for the −0.05 MPa and −0.5 MPa treatments, with average values greater than 0.8. However, the stress produced by the −1 MPa treatment in most cultivars decreased this value; the −2 MPa treatment generated the most significant loss in efficiency for all cultivars. Similarly, the PSII light-adapted maximum efficiency (Fv’/Fm’) was lowest in the −2 MPa treatment (Figure 4B). The values of the electron transfer rate (ETR) presented the same pattern (Figure 4A); however, for this variable, the response of the cultivars to the −0.5 MPa and −1 MPa treatments was very stable, and therefore, the drastic drop is reflected in the comparison between the −0.05 MPa and −2 MPa treatment, showing reductions close to 40% in the ETR.

**Figure 5:**
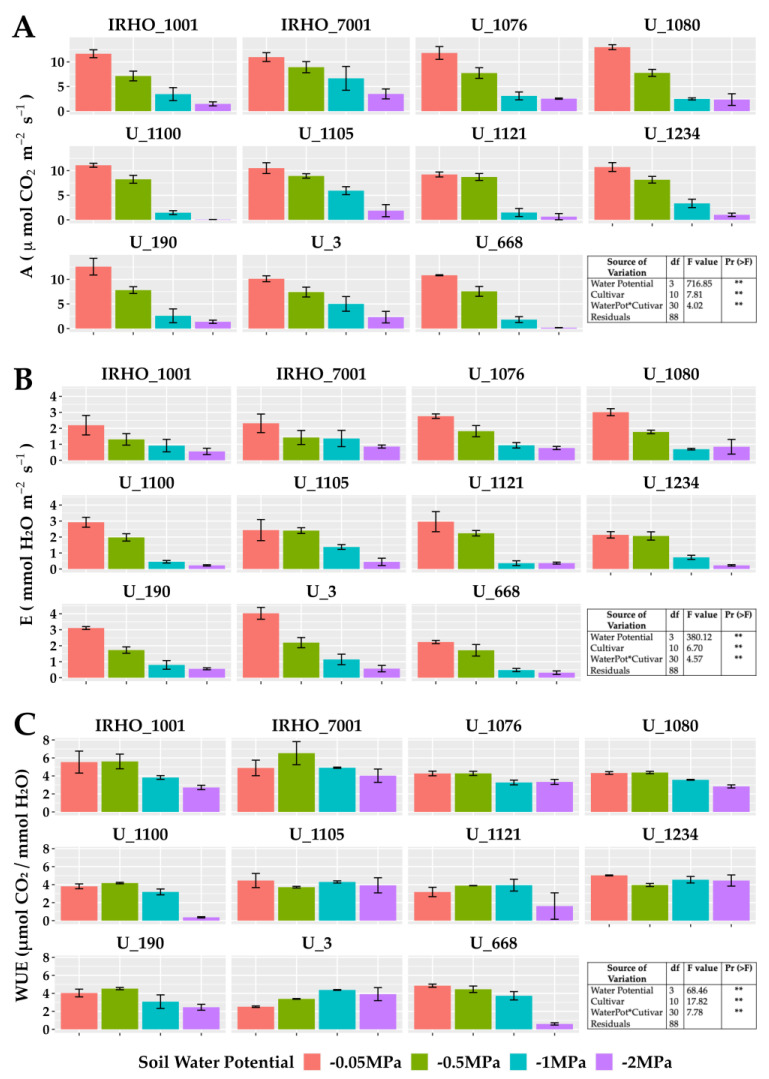
Gas exchange parameters of seedlings of 11 oil palm (*Elaeis guineensis*) cultivars subjected to four soil water potentials (−0.05 MPa, −0.5 MPa, −1 MPa, and −2 MPa) during 60 days of treatment. (**A**) A, photosynthetic rate. (**B**) E, transpiration. (**C**) WUE, water use efficiency of photosynthesis. Each box corresponds to the mean ± SD. (n = 15). The tables in each panel correspond to the two-way ANOVA with significance levels. NS: not significant; ** highly significantly different *p* ≤ 0.01.

In the group of gas exchange variables (Figure 5), the photosynthetic (Figure 5A) and transpiration (Figure 5B) rates, which undoubtedly are among the parameters most affected by water resources in the soil, agree with other studies and show strong decreases. The most affected cultivars in photosynthesis were U 1100, U 1121, U 1234, and U 668, and in transpiration—U 1100, U 1121, and U 1234. The cultivars least affected by the water deficit were U 1076 and IRHO 7001.

### 2.3. Biochemical Parameters

**Figure 6:**
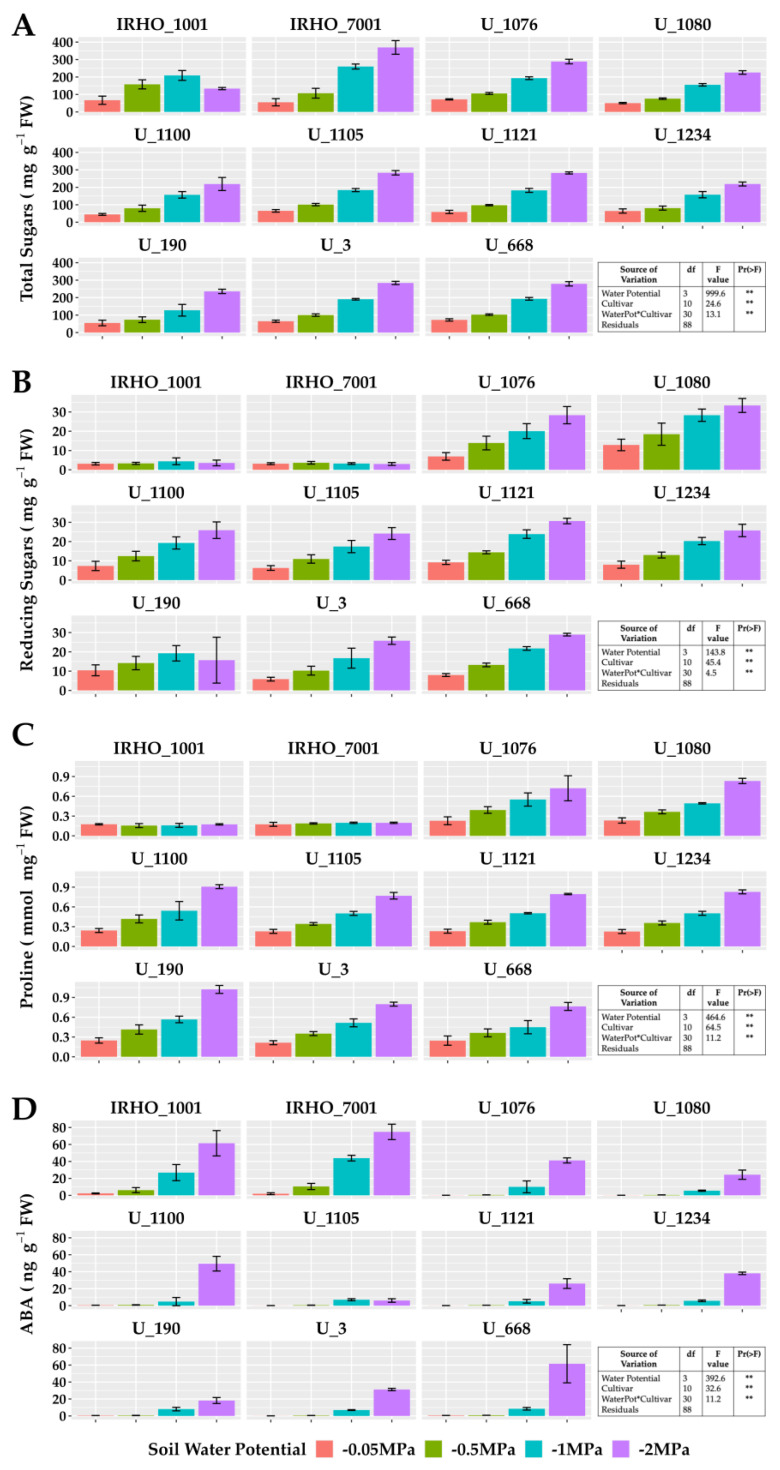
Osmolyte and ABA content of seedlings of 11 oil palm (*Elaeis guineensis*) cultivars subjected to four soil water potentials (−0.05 MPa, −0.5 MPa, −1 MPa, and −2 MPa) during 60 days of treatment. (**A**) Total sugar content. (**B**) Reducing sugar content. (**C**) Proline. (**D**) Abscisic acid. Each box corresponds to the mean ± SD. (n = 15). The tables in each panel correspond to the two-way ANOVA with significance levels. ** highly significantly different *p* ≤ 0.01.

In plants subjected to −2 MPA soil water potential, proline increased up to 300%, except for IRHO cultivars. The same behavior was observed with reducing and total sugars; however, in the latter, cultivar IRHO 7001 showed the most variation, with an increase in the concentration of more than 500% when comparing field capacity conditions and severe stress treatment. Abscisic acid, a hormone closely associated with the water deficit response, showed an accumulation pattern as stress severity increased in all cultivars. However, the hormone concentrations in the IRHO materials under field conditions and mild stress (−0.5 MPa) were the highest among the cultivars, up to 10 times higher than the others (Figure 6).

**Figure 7:**
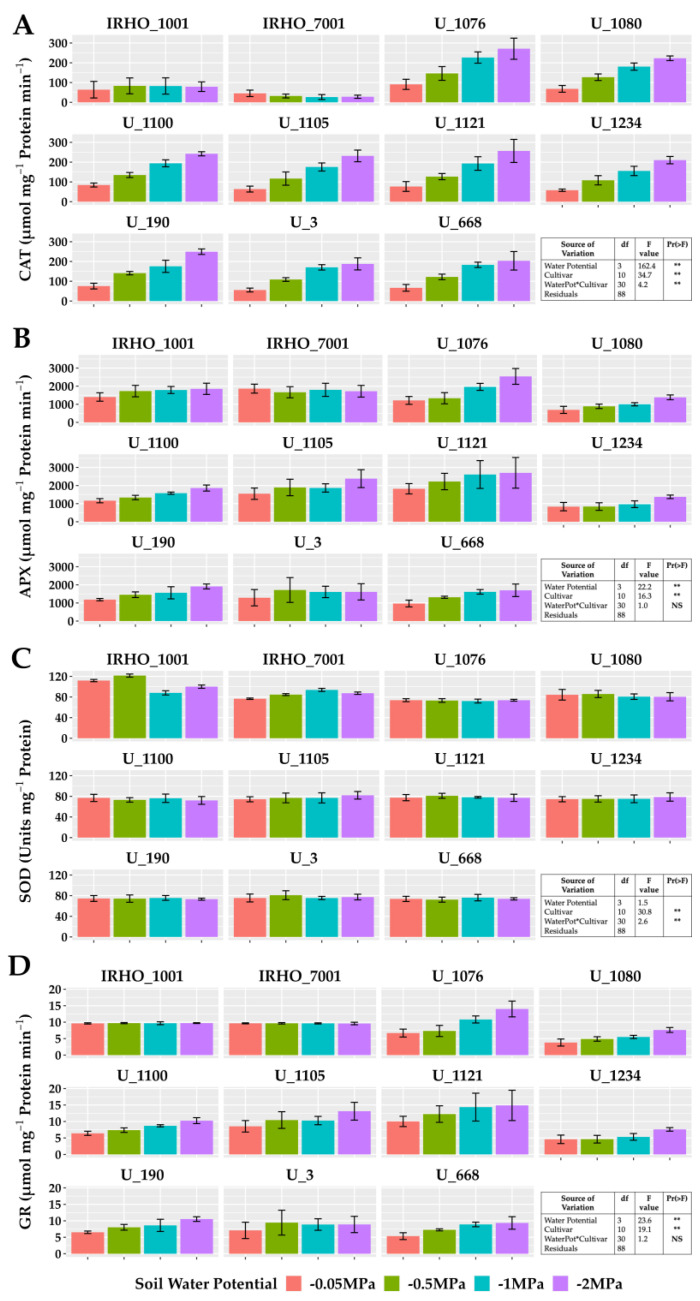
Antioxidant enzyme activity of seedlings of 11 oil palm (*Elaeis guineensis*) cultivars subjected to four soil water potentials (−0.05 MPa, −0.5 MPa, −1 MPa, and −2 MPa) during 60 days of treatment. (**A**) Catalase activity. (**B**) Ascorbate peroxidase activity. (**C**) Superoxide dismutase activity. (**D**) Glutathione reductase activity. Each box corresponds to the mean ± SD. (n = 15). The tables in each panel correspond to the two-way ANOVA with significance levels. NS: not significant; ** highly significantly different *p* ≤ 0.01.

The enzymatic activity of catalase, ascorbate peroxidase, and glutathione reductase showed the same pattern in most cultivars except in the IRHO, showing an increase in activity correlated with the treatments applied, reporting gains of 110% in those associated with GR in cultivar U 1076 between field conditions treatment and severe stress treatment (Figure 7). For catalase, the highest increment was again presented by the cultivar U 1076 and the cultivar U 1234 with 260% more activity compared to the −0.05 MPa and −2 MPa treatments, and within this group, cultivar U 1076 had ascorbate peroxidase increases of 110% in response to the water deficit. The behavior of superoxide dismutase was different, with low variations in response to soil water potentials and without a pattern between treatments. However, the SOD was different among the cultivars, which could indicate a differential potential for responding to the oxidative stress conditions. It should be noted that the activity of CAT, APX, and GR in the IRHO cultivars did not present defined patterns and, in some cases, was very stable across the different treatments (Figure 7).

### 2.4. Principal Component Analysis

**Figure 8:**
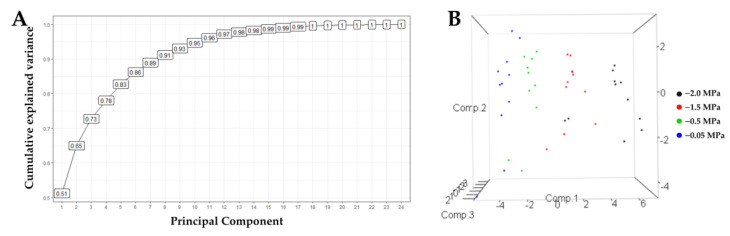
Principal Component Analysis for 22 variables of seedlings of 11 oil palm (*Elaeis guineensis*) cultivars subjected to four soil water potentials (−0.05 MPa, −0.5 MPa, −1 MPa, and −2 MPa) during 60 days of treatment. (**A**) Cumulative variability curve of the sample explained by the consecutive principal components. (**B**). Grouping of cultivars according to three principal components.

The principal component analysis allowed us to understand the different variables’ contribution to the experiment’s variance (Figure 8). A total of 73% of the variance is explained with the first three components and 65% with two. This allowed for the selection of diagnostic variables and showed that the third component contributes 8% to the analysis of the relationship between the vectors of the variables and the cultivars. Thus, we carried out the analyses using two components.

**Figure 9:**
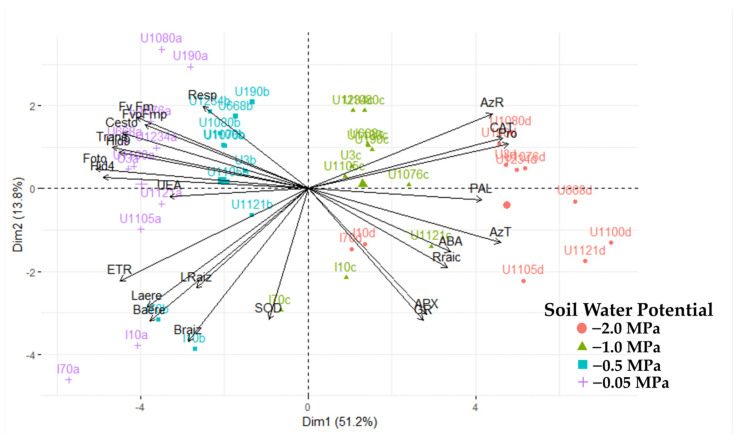
Biplot principal component analysis for 20 variables of seedlings of 11 oil palm (*Elaeis guineensis*) cultivars subjected to four soil water potentials (−0.05 MPa, −0.5 MPa, −1 MPa, and −2 MPa) during 60 days of treatment.

A clear grouping of cultivars was generated, explained by components 1, 2, and 3 (Figure 9), except for the IRHO cultivars, according to the soil’s water stress levels, showing the potential of the variables used against the stressful event. In fact, with 65% explanation (2 components), it was possible to separate the cultivars so that each component’s positive and negative relationships discriminated the differential response of the palms. By relating the response of the cultivars to the four soil water potentials with the variables evaluated, a biplot with two components was generated (Figure 9), where all the cultivars subjected to each treatment were separated by component 1. Within each treatment, they were separated by component 2.

To obtain a more specific view of the interactions of the variables and cultivars in each strain, a PCA was performed in each treatment (Figure 9), and a differential response of the cultivars to each water level in the soil and notable change in the importance of the variables against the response of the plants were found. In the −0.05 MPa treatment, when all plants were grown under good water conditions, the most significant variance was due to growth, osmolyte accumulation, and respiration in both roots and leaves. When plants are subjected to mild stress (−0.5 MPa), the variance is explained by water potential, biomass, ABA, and osmolyte variables. Under moderate stress (−1 MPa), ABA increases as a high source of variation along with antioxidant enzymes and osmolytes, and gas exchange factors are added. When stress is severe (−2 MPa), the most relevant variables are catalase activity, osmolytes, gas exchange, water potential, and ETR.

## 3. Discussion

Water deficit can affect—to different degrees—growth characteristics and metabolic functions because of an interaction between the genotype and the severity of the stress [13,14,21]. Thus, drought tolerance is a complex feature controlled by multiple interacting genes to generate changes in plants’ growth and physiological processes, allowing them to overcome water deficiency [22]. In several species, early detection of drought tolerance has been used to define promising genotypes by exposing young plants to water stress under controlled conditions [23,24] and evaluating different response parameters.

Among the responses related to variations in the growth and development of oil palm plants under water deficit, it has been found that the biomass and length, both in the shoot and the root, are affected by the water content in the soil [18,25]. These results are similar to those found here in the 11 cultivars evaluated under the four soil water potentials, showing that in the biomass and elongation of the shoot and the root, there was a marked difference in biomass between the plants growing in better soil moisture conditions (−0.05 MPa and −0.5 MPa) and treatments with low water availability (−1 MPa and −2 MPa). Henson and Chai [26] developed a study in Malaysia on the root system’s biomass, distribution, and productivity in oil palm. The work was carried out in two zones with contrasting soil moisture; however, they did not report variation in root biomass, possibly because of the short drought periods. However, they found variations in the shoot biomass and the root–stem ratio; something similar was found in this present work and those reported by others. [18,27].

As seen with the growth characteristics, variations in the leaf water potential are observed in the 11 cultivars. Different studies [19,20,21] indicate that cultivars with the least negative leaf water potential, when subjected to a water deficit, are potentially more tolerant to stressful events. Similar conclusions have been found in other monocots, such as sugarcane [28], wheat [24], and corn [29], and dicotyledons, such as cotton [30] and potato [31].

Another critical aspect is related to gas exchange. The rates of photosynthesis and transpiration in oil palm cultivars were, in most cultivars, drastically affected as the available water in the soil decreased, which is a strong response that has been shown before [18] and can discriminate, to a large extent, cultivars that are better at overcoming periods with water limitations [14,32]. On the other hand, an essential gas exchange factor is water used for carbon fixation, which is the relationship between photosynthesis and transpiration. This variable did not present similar patterns among cultivars, which implies a differential response of each cultivar against the water expenditure associated with the need to photosynthesize. It is understood that more efficient water use does not mean greater photosynthesis. The ability of plants to take advantage of the water available in the soil to carry out photosynthesis allows them to maintain their metabolism, as has been reported before [33]. In this same line of variables are those related to chlorophyll fluorescence. According to Ref. [34], values below 0.83 in the Fv/Fm index imply a low photosynthetic yield of the plant and consequently are affected by some stress; however, for Baker [35], the mild water deficit did not affect the primary photochemical events of PSII nor did it modify the associated fluorescence induction parameters, such as Fv/Fm, which is why it is possible that oil palm cultivars exposed to mild and even moderate stress did not show a change. However, when the stress became more severe, there was a drop in the quantum yield, transfer rate (ETR), and Fv/Fm of the 11 cultivars, and the same behavior was reported before [36], suggesting an imbalance between the photochemical and biochemical pathways of photosynthesis, which could potentially lead to the overproduction of ROS in chloroplasts and trigger oxidative damage in cells [20], which is why those cultivars with tolerance can generate mechanisms to reduce the impact, as seen in cotton [30].

Finally, there is the biochemical response, which is aimed at preventing the water deficit from generating a loss of turgor, and changes in the fluidity and composition of the plasma membrane so that plants generally make changes at the cellular and molecular level, such as the accumulation of various osmoregulants and proteins [37] that protect membranes and make the necessary osmotic adjustment to maintain turgor in response to drought. Severe stress caused by water deficit can increase the production of reactive oxygen species (ROS), causing enormous problems in plants [38]. In oil palm, the cultivars in which there are reports of biochemical traits associated with tolerance show an “improved” antioxidant system [12]. It has been shown that POD and SOD activity increases at the beginning of the stressful event to mitigate the damage of the water deficit; however, as the stress period progresses, the conductivity index and relative injuries of the seedlings increase significantly [39]. In our results, CAT, APX, and GR activities are related to the response of the different cultivars to oxidative stress. Thus, these enzymes’ activity increased with the severity of the stress. Interestingly, the changes in these enzyme activities were not present in IHRO 7001 and IHRO 1001, which, based on the multivariate analyses, could be considered the most tolerant to water stress from the 11 tested cultivars, indicating a different or complementary mechanism of tolerance to the stress. In this respect, the consistently high SOD activity, compared to the other cultivars, could play a role in the tolerance of these two cultivars.

Regarding the accumulation of protective osmolytes, it has been shown that some cultivars of oil palm accumulate proline and sugars, thus maintaining turgor in plants subjected to water deficit [16], which is a response that was found in the cultivars evaluated in this work. Additionally, the accumulation of abscisic acid (ABA) seen in the 11 cultivars, especially in the low-moisture soil treatments, may be considered a response to the stressful event since ABA plays a vital role in the adaptive responses to environmental stress such as drought [40] since its function has been seen in the signal transduction pathways between the perceived water stress signal and gene expression [41]. This was seen in palm oil, where two ABA-sensitive regulatory proteins and their role in the ABRE- and DRE/CRT-mediated expression were reported, finding the involvement of the EABF and EABF1 proteins in the stress response and the ABA signaling pathway [42].

As shown above, each of the variables evaluated has been reported in studies with oil palm and associated with water deficit; it is essential to see the contribution that these variables grant to the cultivars defined as tolerant or to be able to categorize them if not. The eight variables that contributed most (according to the cosine squares of the PCA) in broadly differentiating the cultivars are, in order of importance, photosynthesis (0.886), predawn water potential (0.803), proline (0.706), transpiration (0.732), catalase activity (0.725), total sugars (0.708), leaf water potential (0.686), and electron transfer rate (0.678). Supported by the PCA, a clear predominance of Deli × La Mé (IRHOs) cultivars was observed upon separating from the other related cultivars in each treatment, including the field conditions. IRHO are cultivars that, under moderate to severe stress, presented higher photosynthesis and transpiration rates than other cultivars; the water potentials were the least negative both in the predawn and from 9:00 am to 11:00 am. Additionally, these cultivars had a higher electron transfer rate (ETR) and the highest concentrations of sugars. However, in terms of proline accumulation and catalase activity, they were much lower than the other cultivars, which possibly indicates the use of another osmolyte (sugar type) since the IRHOs had the highest concentration of total sugars and the possibility of having a tolerance pathway related to signals mediated by ABA since these cultivars had the highest concentrations of this hormone.

## 4. Materials and Methods

### 4.1. Location

This work was performed in the Palmar de la Vizcaina Experimental Field, Department of Santander—Colombia (6°58 N; 73°42 W). The site is at an altitude of 140 m, has a relative humidity of 75%, and an average temperature of 29 °C. A 20 × 10 × 3 m mesh housing was built for the experiment, with a polycarbonate roof and five mesh walls. Inside the mesh house, the relative humidity was lower than 50%, and the temperature reached 35 °C. To maintain the relative humidity at least at 50% and avoid limitations to photosynthesis, external humidifiers were added, which also contributed to keeping the temperature below 32 °C.

### 4.2. Plant Material

For the evaluation, 11 commercial oil palm cultivars (*Elaeis guineensis*) were selected (Table 1). The germinated seeds were sown in prenursery bags and kept for two months. 

### 4.3. Drought Treatment

The oil palm seedlings were held for one month under field capacity conditions. Then, they were subjected for 60 days to four water stress treatments corresponding to the soil water potential: −0.05 MPa, −0.5 MPa, −1 MPa, and −2 MPa. The soil’s water retention characteristics, including field capacity (FC) and wilting point (WP), were determined to maintain the plants under the corresponding soil water potential. In this study, FC and WP values were approximately 38% and 22%, respectively. The water retention curve and the parameters were used to establish baseline conditions and calculate the daily amount of water that had to be replaced in the containers to ensure the soil water potential of the treatments based on the soil moisture content. SM200 sensors, a Theta Probe, and an equitensiometer (Delta-T, Cambridge, UK) connected to a DL2 data collector (Delta-T, UK) were used to monitor the water content. An automated and monitored irrigation system consisting of 8 L h^−1^ capacity droppers connected to a 3 mm drip hose delivered the necessary water to ensure that the soil water potential corresponded to the defined treatment. Each container where the palms were planted for the water deficit treatments had four droppers strategically positioned in its corners to ensure uniform irrigation coverage and a maximum irrigation depth of 10 cm.

### 4.4. Growth Parameters

Complete plants were taken for destructive sampling. First, the roots were washed with a soft jet of water. Then, with a pruning shear, the aerial part of the roots was separated, and the roots and aerial part were measured with a ruler. Immediately, the fresh weight of the tissues was recorded on a scale; then, they were wrapped in aluminum foil and left in a drying oven at 80 °C for 72 h, and the dry weight of the root and aerial part (leaves and bulb) was recorded.

### 4.5. Physiological Parameters

To quantify gas exchange (photosynthesis, transpiration), a portable photosynthesis meter LI6400XT (LiCor, Inc., Lincoln, NE, USA) was used. The reference parameters of CO_2_ were set at 400 ppm, PAR radiation 1000 μmol m^−2^ s^−1^, block temperature at 30 °C and flow at 170 mmol^−1^, and measurements were performed on leaf number 3 of each plant from 4:00 to 6:00 am to measure respiration and from 9:00 to 11:00 am to measure photosynthesis; chlorophyll fluorescence was determined using an FMSII modulated fluorometer (Hansatech, King’s Lynn, UK), measuring leaf 3 of each palm, and the maximum quantum yield of PSII (Fv/Fm) was measured at night and subsequently light-adapted with actinic lighting for 2 min to measure the quantum yield of PSII (Φ) and electron transfer rate (ETR). Leaf water potential was measured with a pressure chamber (SoilMoisture, Goleta, CA, USA) using palm leaf 3 from 4:00 to 6:00 am (predawn) and 9:00 to 11:00 am.

### 4.6. Biochemical Parameters

From the leaflets that were measured for gas exchange, tissue was collected and preserved in liquid nitrogen; then, in the laboratory, it was macerated with liquid nitrogen, lyophilized, and separated for the quantification of the different variables using the Synergy Mx equipment (BioTek, Winooski, VT, USA), as follows:

Total sugars were determined with 15 mg of fresh leaf tissue [43] at 4 °C. The content was expressed as mg of sugar g^−1^ of fresh tissue. Reducing sugars were determined with 15 mg of fresh leaf tissue [44,45] at 4 °C. The content was expressed as mg of reducing sugar g^−1^ of fresh tissue. The proline was quantified with ninhydrin in an acid medium [46] at 520 nm at 25 °C, expressing the content as mg proline g^−1^ of fresh tissue. The concentration of abscisic acid (ABA) was determined using a Phytodetek.(Agdia, Rikhart, IN, USA) ABA Test Kit [47].

For the antioxidant enzymatic activity, extraction of catalase (CAT, EC.1.11.1.6) and peroxidase (POD, EC 1.11.7.1) was carried out in 250.0 mg of fresh tissue with 50 mM sodium phosphate buffer, 3% (*w*/*v*) of PVP-40, pH 6.80 at 4 °C. The activity of the catalase enzyme (CAT, EC 1.11.1.6) was determined using a permanganometric titration [48]; the specific enzymatic activity was defined as mmol H_2_O_2_ min^−1^ mg^−1^ of protein at 37 °C. The activity of the peroxidase enzyme (POD, EC.1.11.1.7) was determined using the O-dianisidine method [49], and the unit of enzymatic activity was defined as the change in Absorbance at 436 nm expressed in min^−1^ mg^−1^ of protein at 37 °C. The activity of ascorbate peroxidase (APX, EC 1.11.1.11) and glutathione reductase (GR, EC 1.6.4.2) was measured in 500.0 mg of fresh tissue with 50 mM Tris-HCl buffer and ten mM EDTA-Na_2_; pH 7.60, at 4 °C. The enzymatic activity of ascorbate peroxidase was recorded with spectrophotometric monitoring [50]. The unit of enzymatic activity of APX was defined as nmol min^−1^ mg^−1^ of protein at 25 °C. The activity of glutathione reductase was measured using the Yannarelli method [51], and the unit of enzymatic activity was defined as nmol min^−1^ mg^−1^ of protein at 25 °C. The activity of superoxide dismutase (SOD, EC1.15.1.1) was measured in 500 mg of fresh tissue with 100 mM Tris HCl at pH 7.50 at 4 °C, and the activity was determined by the inhibition of the cytochrome c oxidation [52]. The enzymatic activity was defined as the amount of enzyme capable of inhibiting 50% of cytochrome c oxidation mg^−1^ of protein min^−1^ at 560 nm at 25 °C. The total content of soluble proteins was determined by the Bradford method [53].

### 4.7. Statistical Analysis

The experimental design employed a completely randomized design arranged in a factorial structure with three replicates. Each replicate consisted of five plants, resulting in 15 per treatment combinations. The factors investigated were water potential, with four levels (−0.05 MPa, −0.5 MPa, −1 MPa, and −2 MPa), and cultivar, with 11 levels representing different oil palm cultivars (Table 1).

A two-way analysis of variance (ANOVA) was conducted to assess the effects of water potential and cultivar on the measured variables. This statistical approach allowed for examining interactions between the two factors and their individual effects on the observed responses. The ANOVA was performed using R software Version 4.3.0, considering a significance level of α = 0.05.

In addition to ANOVA, a principal component analysis (PCA) was conducted to explore the multivariate relationships among the measured variables and identify underlying patterns or clusters within the dataset. PCA was performed using R software Version 4.3.0, with all 22 variables (Table 2) and 11 cultivars (Table 1) included in the analysis. This technique enabled the reduction of dimensionality while retaining most of the variability present in the original dataset.

## 5. Conclusions

Severe and moderate drought stress negatively affected the photosynthesis, transpiration, growth, and Fv/Fm of the 11 oil palm cultivars. The content of proline and sugars accumulated in most cultivars as a function of water restriction in the same way as what happened with the ROS system enzymes. Six characteristics evaluated (photosynthesis, predawn water potential, proline, transpiration, catalase activity, sugars) were determined to be the most discriminant when selecting oil palm cultivars with tolerance to water deficit. The DTI, PCA, and integrated classification methods showed that the cultivars IRHO 7001 and IRHO 1001 were identified as potentially tolerant to different water stress levels. 

## Figures and Tables

**Table 1:** Parental line (mother and father) of 11 commercial cultivars of *E. guineensis* subjected to water stress.

Cultivar	Mother	Father	Cultivar	Mother	Father
IRHO 1001	Deli	La Mé	U 3	Congo Mixto	Mongana
IRHO 7001	Deli	La Mé	U 1105	(Ekona × Djongo)	Congo Mixto
U 1076	Djongo	Congo Mixto	U 1121	Djongo	Congo Mixto
U 1080	Avros	Djongo	U 1234	(Mongana × Nifor)	Congo Mixto
U 1100	Djongo	Ekona	U 668	Congo Mixto	Mongana
U 190	Djongo	Mongana			

**Table 2:** Variables measured in 11 commercial cultivars of *E. guineensis* subjected to water stress (soil water potential = −0.05 MPa, −0.5 MPa, −1 MPa, and −2 MPa).

Variable	Unit	Variable	Unit
Dry weight root	g	Leaf φ 4 A.M	MPa
Dry weight shoot	g	Leaf φ 9 A.M	MPa
Root length	cm	Proline	mg g^−1^ FW
Height	cm	Total sugars	mg g^−1^ FW
ETR	--	Reducing sugars	mg g^−1^ FW
Fv/Fm	--	ABA	μg g^−1^ FW
Φ	--	POD	Unit min^−1^ mg^−1^ protein
A	µmol CO_2_ m^−2^ s^−1^	APX	nmol min^−1^ mg^−1^ protein
E	mmol H_2_O m^−2^ s^−1^	GR	nmol min^−1^ mg^−1^ protein
DR	µmol CO_2_ m^−2^ s^−1^	CAT	mmol min^−1^ mg^−1^ protein
WUE	µmol CO_2_/mmol H_2_O	SOD	min^−1^ mg^−1^ protein

ETR = electronic transfer rate; Fv/Fm = maximum quantum yield of PSII; Φ = quantum yield of PSII; A = photosynthetic rate measured at saturating irradiance; E = transpiration rate; WUE = water use efficiency of photosynthesis; DR = dark respiration; leaf φ 4 A.M = pre-dawn leaf water potential; leaf φ 9 A.M = actual leaf water potential; ABA = leaf abscisic acid content; POD = leaf peroxydase activity; APX = leaf ascorbate peroxidase activity; GR = leaf glutathione reductase activity; CAT = leaf catalase activity; SOD = leaf superoxide dismutase activity.

## Data Availability

The data presented in this study are available upon request from the corresponding author. Due to privacy restrictions, they are not publicly available.
